# Discovery and Complete Genome Sequence of a Bacteriophage from an Obligate Intracellular Symbiont of a Cellulolytic Protist in the Termite Gut

**DOI:** 10.1264/jsme2.ME16175

**Published:** 2017-03-17

**Authors:** Ajeng K. Pramono, Hirokazu Kuwahara, Takehiko Itoh, Atsushi Toyoda, Akinori Yamada, Yuichi Hongoh

**Affiliations:** 1 Department of Biological Sciences, Tokyo Institute of TechnologyTokyo 152–8550Japan; 2 Department of Biological Information, Tokyo Institute of TechnologyTokyo 152–8550Japan; 3 National Institute of GeneticsShizuoka 411–8540Japan; 4 Division of Marine Biomaterial Science, Graduate School of Fisheries Science and Environmental Studies, Nagasaki UniversityNagasaki 852–8521Japan

**Keywords:** symbiosis, gut bacteria, virus, virome, insect

## Abstract

Termites depend nutritionally on their gut microbes, and protistan, bacterial, and archaeal gut communities have been extensively studied. However, limited information is available on viruses in the termite gut. We herein report the complete genome sequence (99,517 bp) of a phage obtained during a genome analysis of “*Candidatus* Azobacteroides pseudotrichonymphae” phylotype ProJPt-1, which is an obligate intracellular symbiont of the cellulolytic protist *Pseudotrichonympha* sp. in the gut of the termite *Prorhinotermes japonicus*. The genome of the phage, designated ProJPt-Bp1, was circular or circularly permuted, and was not integrated into the two circular chromosomes or five circular plasmids composing the host ProJPt-1 genome. The phage was putatively affiliated with the order *Caudovirales* based on sequence similarities with several phage-related genes; however, most of the 52 protein-coding sequences had no significant homology to sequences in the databases. The phage genome contained a tRNA-Gln (CAG) gene, which showed the highest sequence similarity to the tRNA-Gln (CAA) gene of the host “*Ca.* A. pseudotrichonymphae” phylotype ProJPt-1. Since the host genome lacked a tRNA-Gln (CAG) gene, the phage tRNA gene may compensate for differences in codon usage bias between the phage and host genomes. The phage genome also contained a non-coding region with high nucleotide sequence similarity to a region in one of the host plasmids. No other phage-related sequences were found in the host ProJPt-1 genome. To the best of our knowledge, this is the first report of a phage from an obligate, mutualistic endosymbiont permanently associated with eukaryotic cells.

Bacteriophages are the most abundant biological entities on Earth (30), and are even found in the intracellular bacterial symbionts of eukaryotes (23). The genomes of many commensal *Wolbachia* strains contain WO prophages (18), and a facultative endosymbiont of aphids, “*Candidatus* Hamiltonella defensa”, is often infected with lysogenic phage APSE (29). However, *Wolbachia* strains that have evolved into mutualistic endosymbionts that are permanently associated with filarial nematodes or bed bugs have no or only relics of prophages in their genomes (9, 24). Similarly, phages that infect the primary (ancient, mutualistic) endosymbionts of insects, such as *Buchnera aphidicola*, have yet to be reported (6, 22). Thus, obligate intracellular symbionts that are essential for host survival are apparently free from phage infection.

Termites feed exclusively on dead plant matter, and nutritionally depend on gut microbes, which play crucial roles in the digestion of lignocellulose and provision of nitrogenous compounds (7, 13, 19, 28, 45, 46). This symbiosis is multilayered, with cellulolytic gut protists harboring both endo- and ectosymbiotic bacteria (7, 13). For example, the cellulolytic protist genus *Trichonympha* is always associated with the cytoplasmic symbiont “*Candidatus* Endomicrobium trichonymphae” (27, 38). “*Ca.* E. trichonymphae” are vertically transmitted and have co-diversified with their *Trichonympha* host over more than 40 million years (14, 47). A previous genome analysis suggested that “*Ca.* E. trichonymphae” provides essential nitrogenous compounds to the protist and termite host (12). “*Ca.* E. trichonymphae” and other *Endomicrobium* endosymbionts possess clustered regularly interspaced short palindromic repeats (CRISPR) and associated genes (*cas*) (12, 16, 48), which comprise an adaptive and heritable defense system against foreign DNA (17). At least one CRISPR/Cas system in “*Ca.* E. trichonymphae” phylotype Rs-D17 is functional (16). Thus, *Endomicrobium* endosymbionts may still be subjected to infection by phages despite their long-standing, obligately intracellular lifestyle within a specific host.

Tadmor *et al.* (2011) identified viral marker genes in metagenomic sequences from the gut content of the termite *Nasutitermes* sp., and also in single bacterial cells, sorted using a microfluidic device, from the gut of the termite *Reticulitermes hesperus* (39). The first complete phage genome sequence from a termite gut was recently reported. The phage, CVT22 (family *Podoviridae*), infects *Citrobacter* sp. (43), which is a minor resident of the gut of *Coptotermes* termites (1, 37). No other studies on viruses in the termite gut have been published, and no phages that infect bacterial symbionts of gut protists have been identified.

In the present study, we report the complete genome sequence of a phage obtained during a genome analysis of “*Ca.* Azobacteroides pseudotrichonymphae” (order *Bacteroidales*) that resides within the cells of the cellulolytic protist *Pseudotrichonympha* sp. (phylum *Parabasalia*) in the gut of the termite *Prorhinotermes japonicus*. “*Ca.* A. pseudotrichonymphae” is an obligate intracellular symbiont of *Pseudotrichonympha* species, which are present in the gut of most termite genera in the family Rhinotermitidae. A previous study showed that “*Ca.* A pseudotrichonymphae”, *Pseudotrichonympha* protists, and rhinotermitid termites have co-diversified (26). “*Ca.* A. pseudotrichonymphae” phylotype CfPt1-2 is the predominant bacterial species in the gut of the rhinotermitid termite *Coptotermes formosanus* (25), in which the bacterium fixes dinitrogen and provides amino acids and co-factors (11, 15). Our discovery of a phage that most probably infects the obligate endosymbiont of the *Pseudotrichonympha* protist provides new insights into the multilayered symbiotic relationship in termites.

## Materials and Methods

### Termite collection, DNA extraction, and a 16S rRNA gene cloning analysis

A colony of *P. japonicus* (family Rhinotermitidae) termites was collected from Lanyu Island, Taiwan, in 2012. DNA was extracted from the entire guts of 10 worker termites, as described previously (41), and subjected to PCR amplification of the 16S rRNA gene using the *Bacteria*-specific primers 27F-mix (5′-AGRGTTTGATYMTGGCTCAG-3′) and 1390R (5′-ACGGGCGGTGTGTACAA-3′), as described previously (10, 42). The resulting amplicons were purified and cloned using a TOPO TA Cloning Kit for Sequencing (Invitrogen, Carlsbad, CA, USA) and Competent Quick DH5α *Escherichia coli* (Toyobo, Osaka, Japan) as per the manufacturers’ instructions. Ninety clones were arbitrarily chosen for sequencing on an ABI3730 genetic analyzer (Applied Biosystems, Waltham, MA, USA) using the primers T7 and T3, according to the manufacturer’s instructions.

### Whole genome amplification (WGA)

Single cells of the protist *Pseudotrichonympha* sp. were collected from *P. japonicus* guts using a TransferMan NK2 micromanipulator (Eppendorf, Hamburg, Germany). Each protist cell was disrupted by adding 1% Tween 20 (Nacalai Tesque, Kyoto, Japan) (34), and bacterial cells that leaked out were collected using the micromanipulator and subjected to isothermal WGA by a GenomiPhi HY kit (GE Healthcare, Chicago, IL, USA), as described previously (11). All steps were conducted in a clean-room.

### Genome sequencing and assembly

Sequencing was performed using the Illumina MiSeq platform and MiSeq Reagent Kit v2 (300 cycles), with MiSeq Reagent Kit v3 (600 cycles) being used for a more detailed analysis. Libraries for paired-end and mate-pair sequencing were prepared using a TruSeq DNA PCR-Free Sample Prep Kit and Nextera Mate Pair Sample Prep Kit (Illumina, San Diego, CA, USA), respectively. The generated reads were quality-filtered using Prinseq (36), and then assembled into contigs and scaffolds using SPAdes 3.9.0 (5). Gaps within and between scaffolds were closed by PCR amplification and sequenced using the ABI3730 Genetic Analyzer. In addition, sequence reads of 4–5 kb were generated on the PacBio RSII platform using a BluePippin DNA Size-Selection system (Sage Science, Beverly, MA, USA) and P6-C4 chemistry (Pacific Biosciences, Menlo Park, CA, USA). PacBio reads were used to verify the assembly and circularity of the scaffolds by mapping the reads using the BLASTn program of BLAST+ suite (8). MiSeq-read coverage was calculated by counting *k*-mers covering each scaffold in the assembly using SPAdes 3.9.0.

### Gene prediction and annotation

Gene predictions and homology-based predictions of gene functions were conducted using the RAST (3) and METAVIR servers (33). tRNA sequences were predicted using tRNAscan (35) and the tRNA Gene DataBase Curated by Experts (2). Codon usage was calculated from protein-coding nucleotide sequences (CDSs) using EMBOSS ver. 6.4.0 (32). The annotation was manually curated using BLASTp searches against the NCBI non-redundant (nr) protein sequence database (ftp://ftp.ncbi.nlm.nih.gov/blast/db/) and refseq viral database (ftp://ftp.ncbi.nlm.nih.gov/refseq/release/viral/) with a cut-off e-value <10^−3^. We identified contigs derived from DNA viruses using a keyword (*virus, virion, prophage, terminase, capsid, head, tail, fiber, baseplate, portal, lysis, structural, T4, lambda, mu, lambdoid, podo*, myovir*, siphovir*, integrase, transposase) search of BLASTp results (20). CDSs that were predicted to code for hypothetical proteins were subjected to further BLASTp searches against the NCBI conserved domain database (https://www.ncbi.nlm.nih.gov/Structure/cdd/wrpsb.cgi). Phylogenetic analyses were conducted using MEGA 6.0 (40).

### Database accession numbers

The sequence data obtained in this study were submitted to the DDBJ under the following accession numbers: AP017903 (ProJPt-Bp1-PJA1 genome), BDLX01000001–19 (ProJPt-Bp1-PJA2 draft genome), BDLY01000001-28 (ProJPt-Bp1-PJB1 draft genome), BDLZ01000001-28 (ProJPt-Bp1-PJB2 draft genome), AP017913–9 (ProJPt-1 chromosomes and plasmids), and LC198325–68 (16S rRNA genes).

## Results and Discussion

### Reconstruction of the genome of the host “*Ca.* A. pseudotrichonymphae” phylotype ProJPt-1

Four WGA samples, each prepared from bacterial cells contained in a single *Pseudotrichonympha* cell, were subjected to genome sequencing. These WGA samples were designated PJA1, PJA2, PJB1, and PJB2, with PJA1 and PJA2 being derived from one termite and PJB1 and PJB2 from another. Sample PJA1 was deeply sequenced, yielding 1,256,368 paired-end reads and 1,557,412 (3–5 kb insert length) and 1,576,369 (5–7.5 kb insert length) mate-pair reads. By using the PJA1 sample, we reconstructed the complete genome sequence of “*Ca.* A. pseudotrichonymphae” phylotype ProJPt-1, comprising two circular chromosomes and five circular plasmids (Table 1, Fig. S1 and S2). This is the second published complete genome of “*Ca.* A. pseudotrichonymphae”, following that of phylotype CfPt1-2 (genomovar CFP2) from the gut of *C. formosanus* (11). The phylogenetic relationship between phylotypes CfPt1-2 and ProJPt-1 was inferred based on the 16S rRNA gene sequences (Fig. S3). The results of the 16S rRNA gene cloning analysis showed that 36 out of 90 clones (40%) from the bacterial gut microbiota of *P. japonicus* workers were phylotype ProJPt-1 (data not shown). This result suggested that phylotype ProJPt-1 is a dominant species in the *P. japonicus* gut, and appears to play a critical role in the symbiotic relationship, as observed for phylotype CfPt1-2 (11, 25).

Each of the two ProJPt-1 chromosomes contained an rRNA gene set. The rRNA genes in chromosome 2 formed a canonical rRNA operon, whereas the operon was separated into two parts in chromosome 1 (Fig. S4). A separated rRNA gene set was also found on the single chromosome of phylotype CfPt1-2 (11). Massive rearrangements were observed between the chromosomes of phylotypes CfPt1-2 and ProJPt-1 (Fig. S5), whereas no mobile elements or restriction-modification systems were found in either genome, unlike in *Endomicrobium* endosymbionts (16, 48). The total size of the two chromosomes (908,378 bp) of ProJPt-1 was markedly smaller than the chromosome of CfPt1-2 (1,114,206 bp). The five plasmids of ProJPt-1 (designated pAPPJ1–5) contained regions with high sequence similarities to certain regions of three out of the four plasmids of CfPt1-2 (pCFPG1, 2, and 3; data not shown). In addition, we identified two contigs (2,275 bp and 1,871 bp) with only one- or two-fold MiSeq-read coverage that contained regions sharing 68–73% nucleotide sequence identities with plasmid pCFPG4 of CfPt1-2. These two contigs may represent a plasmid that is only rarely contained in the ProJPt-1 genome (data not shown). Other detailed information on the ProJPt-1 genome will be published elsewhere.

### Discovery and reconstruction of the phage genome

We recovered one large scaffold (99,643 bp) from the reconstruction of the ProJPt-1 genome, which showed almost no sequence similarity to the genome of phylotype CfPt1-2. The GC content of the scaffold was 42.3%, which differed greatly from that of the chromosomes and plasmids of ProJPt-1 (21.3–28.1%). The only region that shared a high nucleotide sequence identity with the CfPt1-2 genome was a full-length tRNA gene (74 bp). This tRNA-Gln (CAG) gene had the highest sequence similarity (85%) with tRNA-Gln (CAA) of CfPt1-2 (CFPG_tRNA22) in a BLASTn search against the NCBI nr nucleotide sequence database, and showed higher sequence similarity (91%) to the tRNA-Gln (CAA) gene from ProJPt-1 chromosome 1 obtained in this study. A tRNA gene on a virus genome has been used in order to identify the host organism (30). In addition to the tRNA gene, a 472-bp non-coding region of the scaffold shared 90% nucleotide sequence identity with a region of plasmid pAPPJ3 of ProJPt-1.

It was not possible to connect this scaffold by PCR amplification, mate-pair reads, or PacBio reads to any of the ProJPt-1 genome components, and it instead had overlapping terminal sequences. This indicated that the scaffold formed a circular or circularly permuted structure. This circularity was verified by Sanger sequencing of PCR amplification products and by the mapping of PacBio reads. Although this scaffold almost exclusively encoded genes for hypothetical proteins, the METAVIR server, an automatic annotator specialized for viral sequence reads or contigs, identified six viral genes on the scaffold. Five of these genes showed low to moderate degrees of amino acid sequence similarity to genes belonging to phages in the family *Podoviridae* (order *Caudovirales*), while the final gene was similar to a gene from an unclassified viral group (Tables 2 and S1; see below for details on these genes). Taken together, we concluded that this scaffold was the circular or circularly permuted genome of a bacteriophage from the order *Caudovirales*, and strongly suggest that the phage, designated ProJPt-Bp1, infects “*Ca.* A. pseudotrichonymphae” phylotype ProJPt-1. Since the MiSeq-read coverage for the ProJPt-Bp1 genome was similar to those for the two ProJPt-1 chromosomes (Table 1), and because the draft genomes of ProJPt-Bp1 were recovered from the three other samples (PJA2, PJB1, and PJB2) (Table 1 and Fig. S6), the ProJPt-Bp1 phage appeared to be common to the *Pseudotrichonympha*-*Azobacteroides* symbiotic system in the *P. japonicus* gut.

### Characteristics of the ProJPt-Bp1 phage genome

**Fig. 1:**
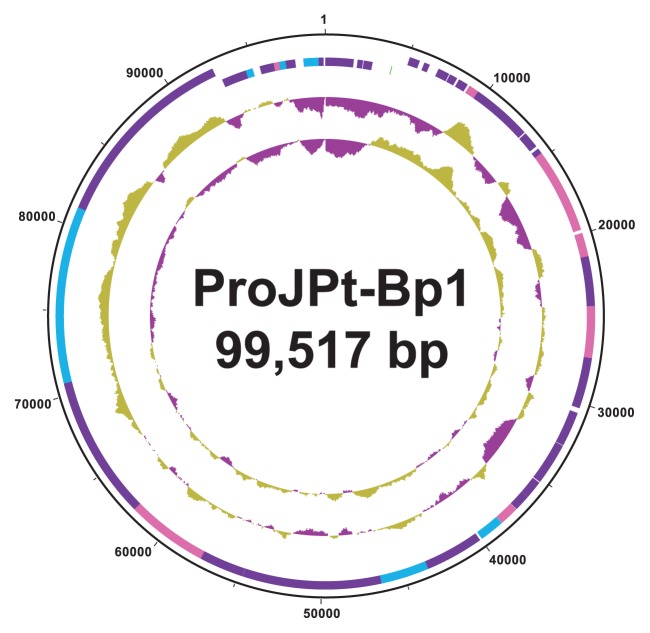
Circular representation of the genome of phage ProJPt-Bp1. Concentric rings denote the following features (from the outer to inner rings): nucleotide positions; protein coding sequences on the forward strand (+) and reverse strand (−) (green: tRNA gene; purple: hypothetical proteins; blue: genes with predicted functions based on the NCBI nr or refseq databases; pink: genes with predicted functions based on the NCBI conserved domain database); G+C content (purple: <50%; gold >50%); GC skew (G−C)/(G+C) (purple: −; gold: +).

The circularly assembled genome of the ProJPt-Bp1 phage was 99,517 bp, with no gaps or ambiguous sites (Fig. 1). The genome encoded 52 putative CDSs and the tRNA-Gln (CAG) gene, with 94% coding density (Table 2 and S1). The genome showed no significant sequence similarity to the genome of the phage CVT22, isolated from the gut of *C. formosanus* (43). The PJPA_051 gene, coding for a putative exonuclease VIII, showed the highest amino acid sequence similarity (31%) to a hypothetical protein (CFPG_P2-6) from plasmid pCFPG2 of CfPt1-2. No homologous gene was identified in the genome of the host, ProJPt-1. A gene coding for a conserved hypothetical protein (PJPA_020), along with three genes for putative structural proteins (PJPA_027, 032, and 041), shared low amino acid sequence identities (24–27%) with the genes of *Cellulophaga* phage phi14:2 (NC_021806) (*Podoviridae*). A gene coding for a protein inside capsid D (PJPA_049) showed 39% amino acid sequence identity to a gene from *Cronobacter* phage Dev2 (NC_023558) (Table 2) and was also phylogenetically related to genes from other *Podoviridae* phages (Fig. S7A).

The ProJPt-Bp1 genome encoded deoxyuridine 5′-triphosphate nucleotidohydrolase (dUTPase: Dut) (PJPA_046), which is present in a wide range of viral genomes (31). ProJPt-Bp1 *dut* showed moderate amino acid sequence similarity (46%) to the *dut* gene of *Mollivirus sibericum* (NC_027867), which belongs to an unclassified dsDNA virus group (Fig. S7B). The Dut enzyme hydrolyzes dUTP to dUMP and pyrophosphate, thereby preventing uracil incorporation into DNA by decreasing the dUTP/dTTP ratio during phage genome replication (31). In addition, it has recently been demonstrated that Dut acts as a regulatory protein that controls various host functions, including the immune system (31).

The tRNA-Gln (CAG) gene from the ProJPt-Bp1 genome was consistently found in the three other draft genomes (from samples PJA2, PJB1, and PJB2) with no sequence variation (Fig. S6). The presence of a tRNA gene is not rare in phage genomes (30); it has been hypothesized that viral tRNA compensates for differences in codon usage bias between a phage and its bacterial host (4). The frequency of CAG codon usage for glutamine was 52.6% in the ProJPt-Bp1 phage genome, whereas it was only 15.1% and 22.1% in the ProJPt-1 and CfPt1-2 chromosomes, respectively (Table S2). Interestingly, the ProJPt-1 genome had no tRNA-Gln (CAG) gene, and reads that did not map to the ProJPt-1 genome also did not contain any tRNA genes with high similarity to the tRNA-Gln (CAG) gene (CFPG_tRNA9) on the CfPt1-2 chromosome. Thus, the presence of the tRNA-Gln (CAG) gene in the phage genome may be advantageous for phage propagation. Alternatively, the tRNA gene may be the attachment site required for the phage to integrate into the host genome. However, such lysogenic phages generally have only a small fragment that corresponds to a part of the host tRNA gene that functions as the attachment site, not a full-length tRNA gene (4). In addition, we did not find any contigs that showed homology between the host and phage genomes, and no integrase gene was identified in the phage genome.

**Fig. 2:**
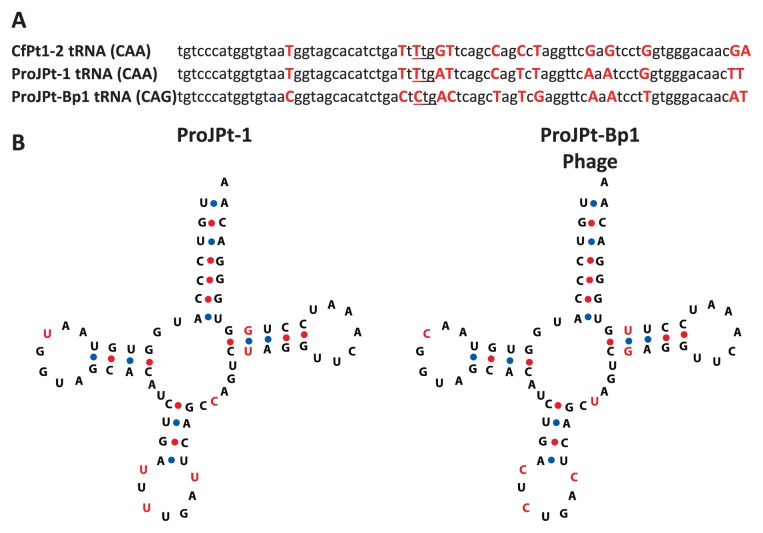
tRNA gene in the ProJPt-Bp1 genome. (A) Alignment of the tRNA gene from phage ProJPt-Bp1 with the homologous tRNA genes from “*Ca.* Azobacteroides pseudotrichonymphae” phylotypes ProJPt-1 and CfPt1-2. The anticodon position is underlined. (B) Predicted secondary structures of tRNA-Gln.

The ProJPt-1 and CfPt1-2 tRNA-Gln (CAA) genes were the most similar to the phage tRNA-Gln (CAG) gene, as described above, indicating that a single nucleotide substitution changed the codon for tRNA-Gln from CAA to CAG in the phage genome (Fig. 2). It is tempting to hypothesize that the ProJPt-Bp1 phage contributes to the host, ProJPt-1, by providing the missing tRNA, although it is difficult to experimentally prove this hypothesis in these uncultured microorganisms. However, based on the predicted secondary structure, the tRNA of the phage appeared to be functional (Fig. 2).

In general, it is difficult for a phage to infect an obligate intracellular bacterium with no free-living phase because the phage must penetrate multiple barriers, *i.e.*, the eukaryotic cell membrane as well as the bacterial cell wall and membranes. Although obligate intracellular symbionts tend to lack a cell wall, as predicted from the genome sequences of “*Ca.* A. pseudotrichonymphae” phylotypes CfPt1-2 (11) and ProJPt-1 (data not shown), this also means that these endosymbionts lack molecules on the cell wall or outer membrane that are recognized by phages for specific infection (44). This appears to be the first report of a phage that infects an obligate, mutualistic, intracellular symbiont permanently associated with its eukaryotic host. It is conceivable that phages are ingested with wood particles by the cellulolytic protist, thereby coming into contact with *Azobacteroides* endosymbionts, as discussed previously for “*Ca.* E. trichonymphae” hosted by *Trichonympha* protists (16, 48). Nevertheless, unlike the *Endomicrobium* endosymbionts, no CRISPR/Cas systems or restriction-modification systems were found in the genomes of “*Ca.* A. pseudotrichonymphae” phylotypes ProJPt-1 and CfPt1-2. Other known defense mechanisms such as abortive-infection systems were also not identified. The apparent absence of defense systems against phages is enigmatic, considering the importance of “*Ca.* A. pseudotrichonymphae” in the termite gut ecosystem. This ProJPt-Bp1 phage may be temperate, perhaps maintained as a plasmid-like entity, and only cause mild damage to the host *Azobacteroides* population. In any case, we need to consider a more complex, multilayered symbiotic system, including phages, in addition to protists, bacteria, and archaea.

## Supplementary material



## Figures and Tables

**Table 1:** Genome features of “*Ca.* Azobacteroides pseudotrichonymphae” phylotype ProJPt-1 and the ProJPt-Bp1 phage.

	Size (bp)	Coverage*	%GC	rRNA	tRNA
ProJPt-1					
Chromosome 1	774,998	250	27.5	3	28
Chromosome 2	139,924	192	28.1	3	11
pAPPJ1 [plasmid 1]	26,809	385	22.2	—	—
pAPPJ2 [plasmid 2]	21,972	1299	27.0	—	—
pAPPJ3 [plasmid 3]	20,721	521	26.6	—	—
pAPPJ4 [plasmid 4]	18,962	662	24.4	—	—
pAPPJ5 [plasmid 5]	13,523	1034	21.3	—	—

Phages					
ProJPt-Bp1 (PJA1)	99,517	224	42.3	—	1
ProJPt-Bp1 (PJA2)**	96,361	—	42.6	—	1
ProJPt-Bp1 (PJB1)**	96,379	—	42.5	—	1
ProJPt-Bp1 (PJB2)**	96,945	—	42.5	—	1

* *k*-mer coverage for the largest *k*-value (127 bp).

** Draft genomes, assembled after retrieving reads mapped to the ProJPt-Bp1 genome of sample PJA1 using Bowtie 2 (21).

**Table 2:** List of genes with predicted functions or domains in the genome of the ProJPt-Bp1 phage.

Gene_id	Predicted domains or functions
PJPA_tRNA1	tRNA-Gln (CAG)
PJPA_011	ND2 superfamily protein
PJPA_015	Cadherin-like beta sandwich domain protein
PJPA_016	Major outer envelope glycoprotein
PJPA_018	DUF4417-cotaining protein
PJPA_026	VIP2; Actin-ADP-ribosylating toxin family protein
PJPA_027*	Structural protein
PJPA_032*	Structural protein
PJPA_036	Epstein-Barr virus nuclear antigen 3B domain protein
PJPA_041*	Structural protein
PJPA_046*	Deoxyuridine 5′-triphosphate nucleotidohydrolase
PJPA_048	Solute carrier families 5 and 6-like protein
PJPA_049*	Phage protein inside capsid D
PJPA_051	Exonuclease VIII

* Phage-related genes identified by the METAVIR server.
