# Supplementary material for: Discovery and Complete Genome Sequence of a Bacteriophage from an Obligate Intracellular Symbiont of a Cellulolytic Protist in the Termite Gut
Source: Microbes Environ. 2017 Mar 17;32(2):112–7. doi: 10.1264/jsme2.ME16175 (PMC5478533; doi:10.1264/jsme2.ME16175)
Supplement: Supplementary file 1 [file 32_112_s1.pdf]

Legends to Supplemental Figures

Fig. S1. Circular representation of two chromosomes from "*Ca*. Azobacteroides pseudotrichonymphae" phylotype ProJPt-1. Concentric rings denote the following features (from outer to inner rings): nucleotide positions starting from base 1 of *dnaA* in chromosome 1, and the predicted *oriC* in chromosome 2; protein-coding sequences (CDSs) (dark blue: CDSs on the forward strand (+), light blue: CDSs on the reverse strand (−), orange: rRNA genes, green: tRNA genes); G + C content (purple: <50%, gold: >50%); GC skew (G − C)/(G + C) (purple: −, gold: +).

Fig. S2. Circular representation of five plasmids of "*Ca.* Azobacteroides pseudotrichonymphae" phylotype ProJPt-1. The concentric rings denote the following features (from outer to inner rings): nucleotide position starting from base 1 of *repA*; protein-coding sequences (CDSs) (dark blue: CDSs on the forward strand [+], light blue: CDSs on the reverse strand [-]); G + C content (purple, < 50%; gold > 50%); GC skew (G − C)/(G + C) (purple, −; gold, +).

Fig. S3. Phylogenetic position of phylotype ProJPt-1 in "*Ca.* Azobacteroides pseudotrichonymphae" based on 16S rRNA gene sequences. A maximum likelihood tree was constructed using the GTR+Γ+I nucleotide substitution model. Bootstrap confidence values (500 resamplings) are indicated on the internal branches. *Paludibacter propionicigenes* WB4, *Bacteroides ovatus* ATCC 8483, and *Bacteroides fragilis* BOB25 were used as outgroups.

Fig. S4. rRNA genes on ProJPt-1 chromosomes. Putative breakpoints of the rRNA operon are shown in yellow wedges. Each of the green, blue, and orange broken lines represents identical intergenic regions between chromosomes 1 and 2.

Fig. S5. Synteny analysis between ProJPt-1 chromosomes and the CfPt1-2 chromosome. Red lines indicate similarly-orientated corresponding regions, while blue lines indicate regions that are oriented in opposite directions. The analysis was conducted using the Artemis Comparison Tool (http://www.sanger.ac.uk/science/tools/artemis-comparison-tool-act).

Fig. S6. Alignment of the ProJPt-Bp1 phage genome from sample PJA1 with draft genomes of ProJPt-Bp1 from samples PJA2, PJB1, and PJB2. Yellow wedges indicate tRNA gene loci. Red lines show syntenic regions. The analysis was conducted using the Artemis Comparison Tool (http://www.sanger.ac.uk/science/tools/artemis-comparison-tool-act). An ~3,100-bp region surrounding nucleotide position 14,000, which contains a gene for a cadherin-like beta sandwich domain protein (PJPA_015), was unique to the ProJPt-Bp1 genome from sample PJA1.

Fig. S7. Neighbor-joining trees of phage-related genes based on deduced amino acid sequences. (A) Protein inside capsid D (111 amino acids); (B) dUTPase (114 amino acids). Sequences were aligned using ClustalW with a gap opening penalty of 10.0 and gap extension penalty of 0.2. The Dayhoff amino acid substitution model was used. An asterisk in panel (A) indicates the sequence from *Mollivirus sibericum*, the taxonomic assignment of which was unclear.

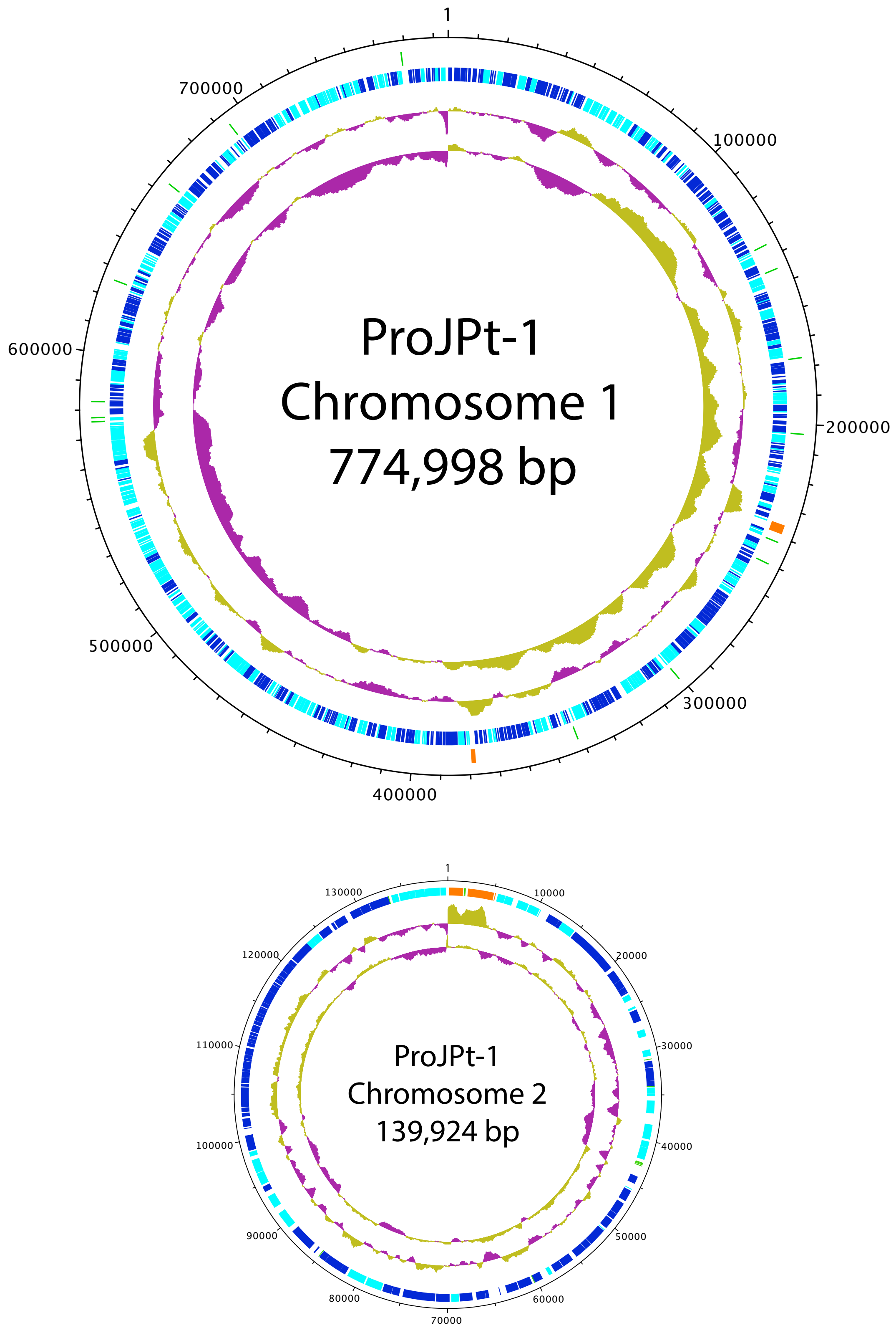

Fig. S1. Circular representation of two chromosomes from "*Ca*. Azobacteroides pseudotrichonymphae" phylotype ProJPt-1. Concentric rings denote the following features (from outer to inner rings): nucleotide positions starting from base 1 of *dnaA* in chromosome 1, and the predicted *oriC* in chromosome 2; protein-coding sequences (CDSs) (dark blue: CDSs on the forward strand (+), light blue: CDSs on the reverse strand (−), orange: rRNA genes, green: tRNA genes); G + C content (purple: <50%, gold: >50%); GC skew (G − C)/(G + C) (purple: −, gold: +).

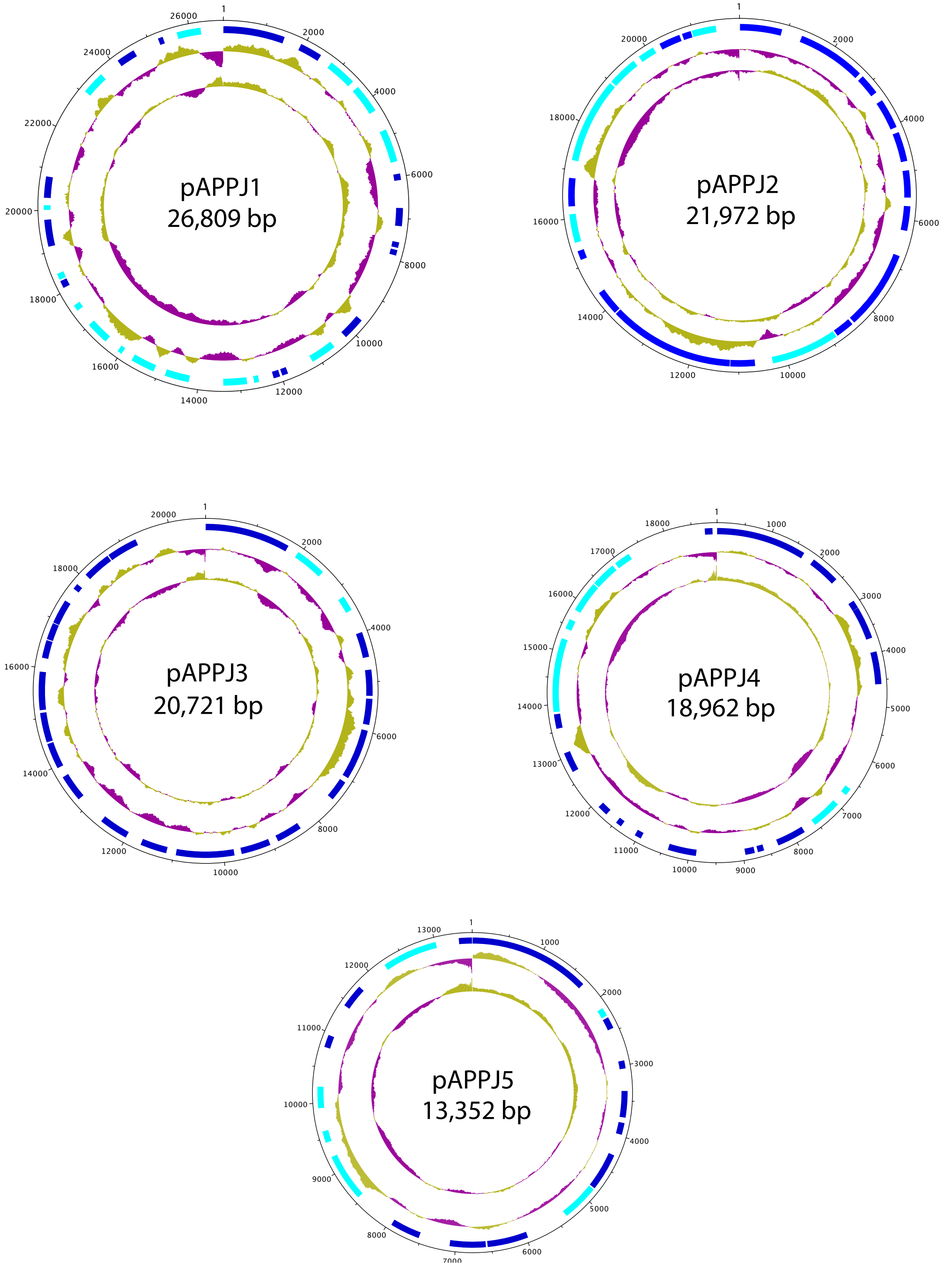

Fig. S2. Circular representation of five plasmids of "*Ca*. Azobacteroides pseudotrichonymphae" phylotype ProJPt-1. The concentric rings denote the following features (from outer to inner rings): nucleotide position starting from base 1 of *repA*; protein-coding sequences (CDSs) (dark blue: CDSs on the forward strand [+], light blue: CDSs on the reverse strand [-]); G + C content (purple, < 50%; gold > 50%); GC skew (G − C)/(G + C) (purple, −; gold, +).

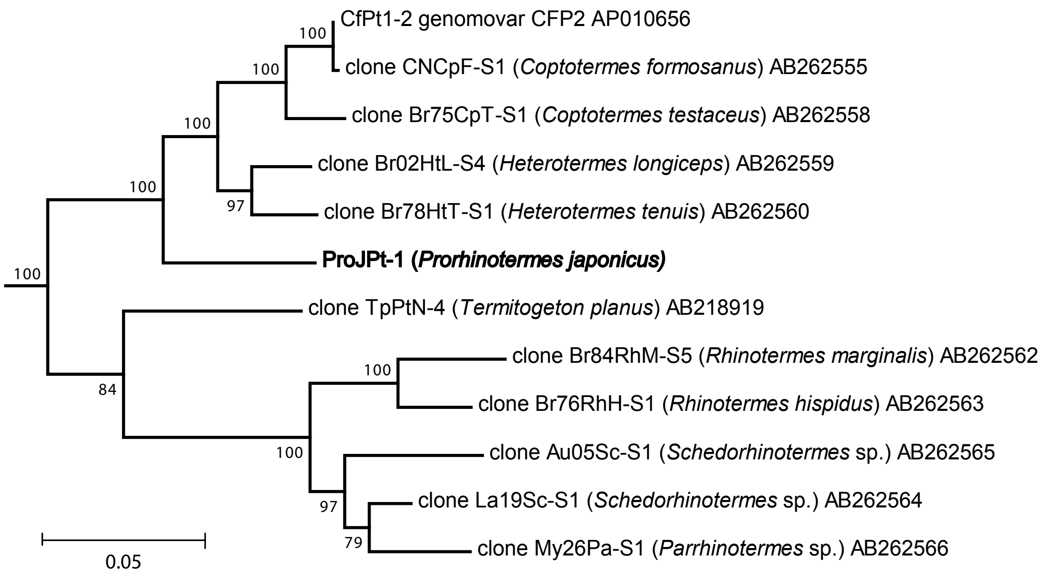

Fig. S3. Phylogenetic position of phylotype ProJPt-1 in "*Ca*. Azobacteroides pseudotrichonymphae" based on 16S rRNA gene sequences. A maximum likelihood tree was constructed using the GTR+Γ+I nucleotide substitution model. Bootstrap confidence values (500 resamplings) are indicated on the internal branches. *Paludibacter propionicigenes* WB4, *Bacteroides ovatus* ATCC 8483, and *Bacteroides fragilis* BOB25 were used as outgroups.

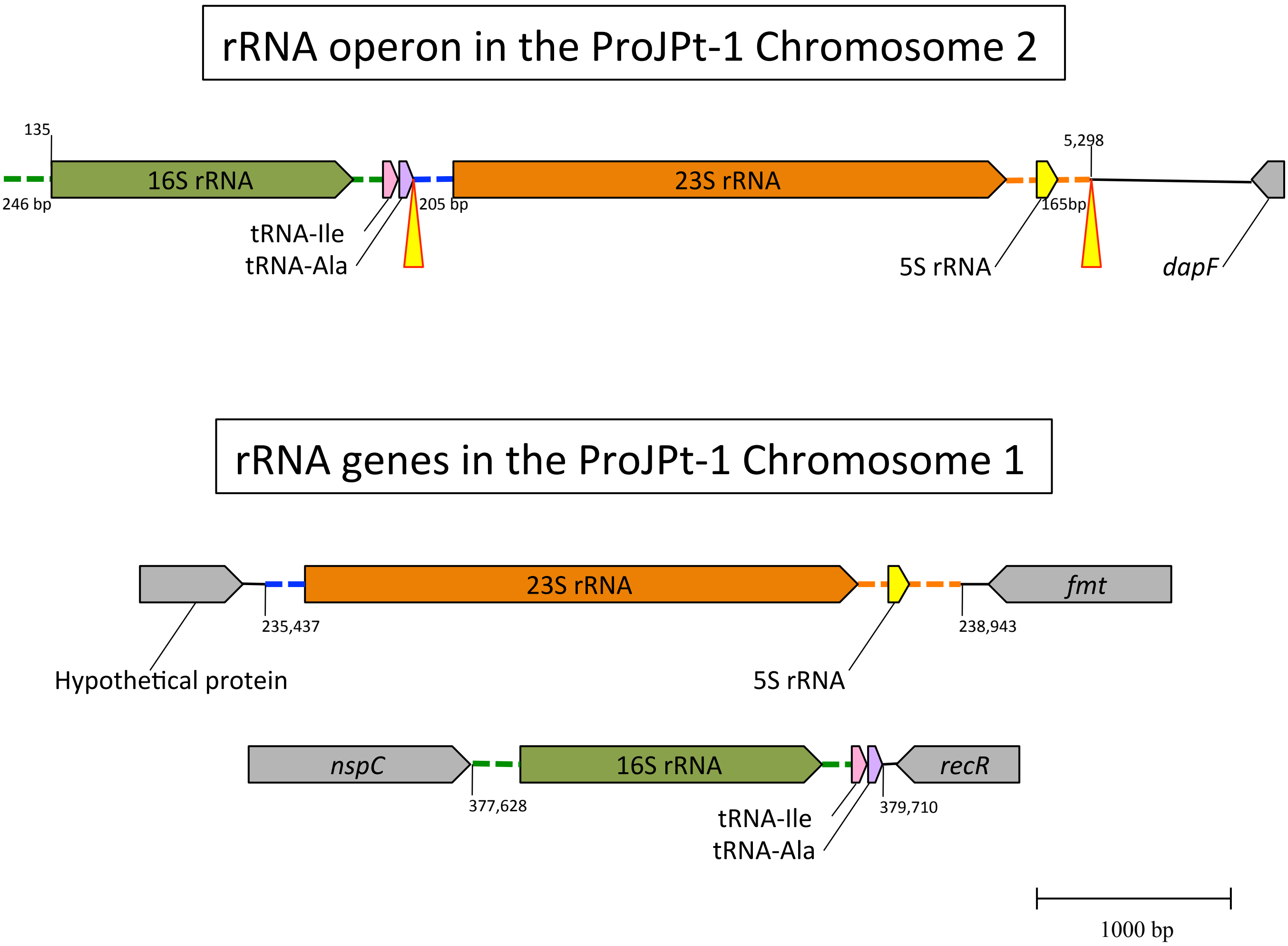

Fig. S4. rRNA genes on ProJPt-1 chromosomes. Putative breakpoints of the rRNA operon are shown in yellow wedges. Each of the green, blue, and orange broken lines represents identical intergenic regions between chromosomes 1 and 2.

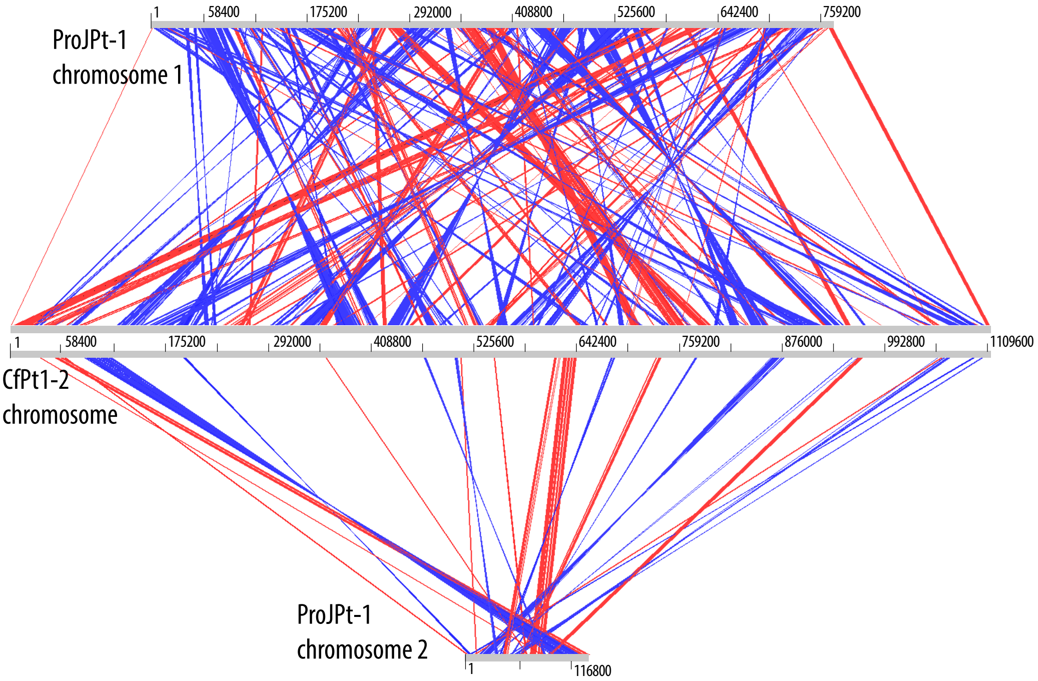

Fig. S5. Synteny analysis between ProJPt-1 chromosomes and the CfPt1-2 chromosome. Red lines indicate similarly-orientated corresponding regions, while blue lines indicate regions that are oriented in opposite directions. The analysis was conducted using the Artemis Comparison Tool (http://www.sanger.ac.uk/science/tools/artemis-comparison-tool-act).

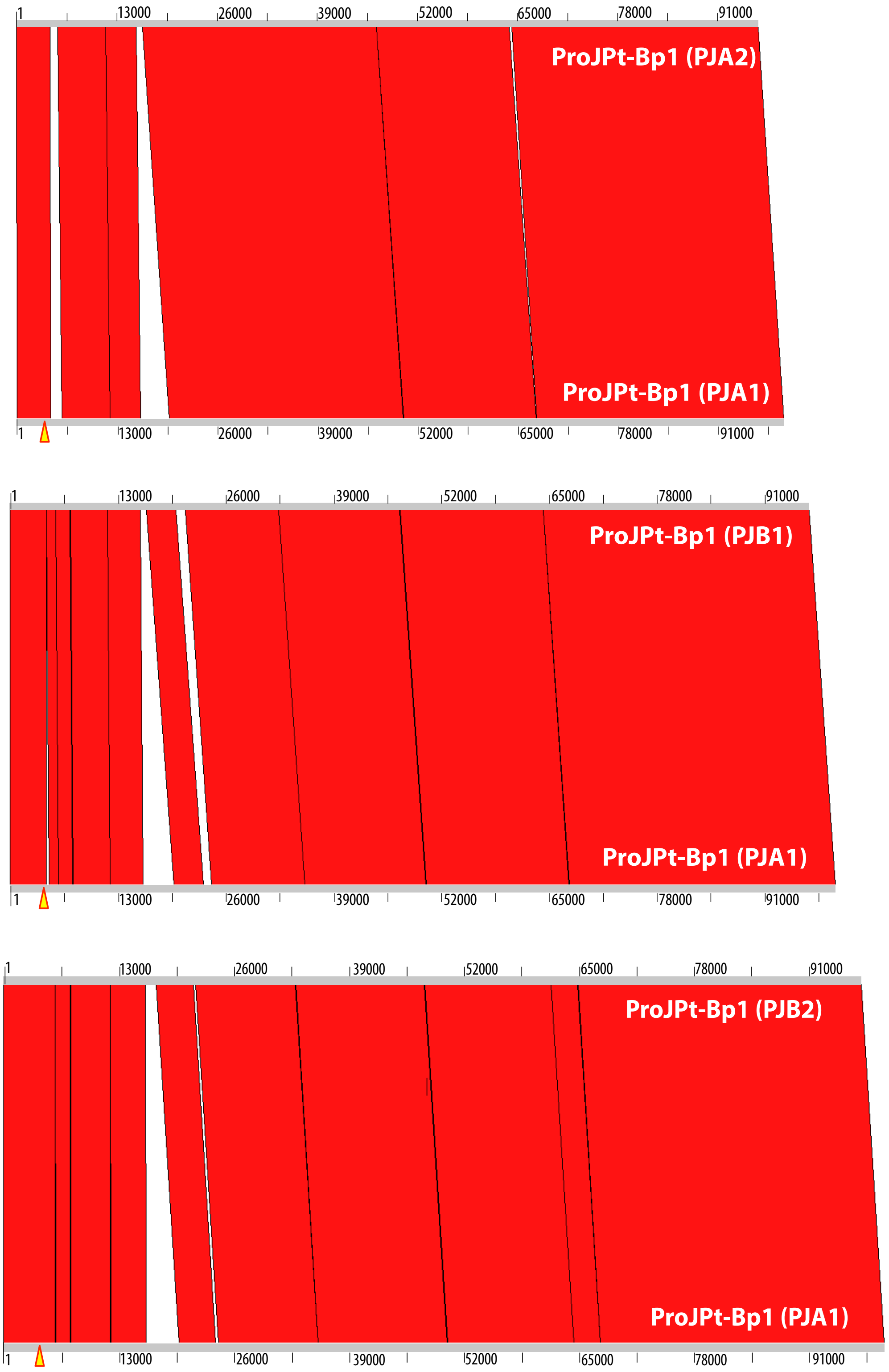

Fig. S6. Alignment of the ProJPt-Bp1 phage genome from sample PJA1 with draft genomes of ProJPt-Bp1 from samples PJA2, PJB1, and PJB2. Yellow wedges indicate tRNA gene loci. Red lines show syntenic regions. The analysis was conducted using the Artemis Comparison Tool (http://www.sanger.ac.uk/science/tools/artemis-comparison-tool-act). An ~3,100-bp region surrounding nucleotide position 14,000, which contains a gene for a cadherin-like beta sandwich domain protein (PJPA_015), was unique to the ProJPt-Bp1 genome from sample PJA1.

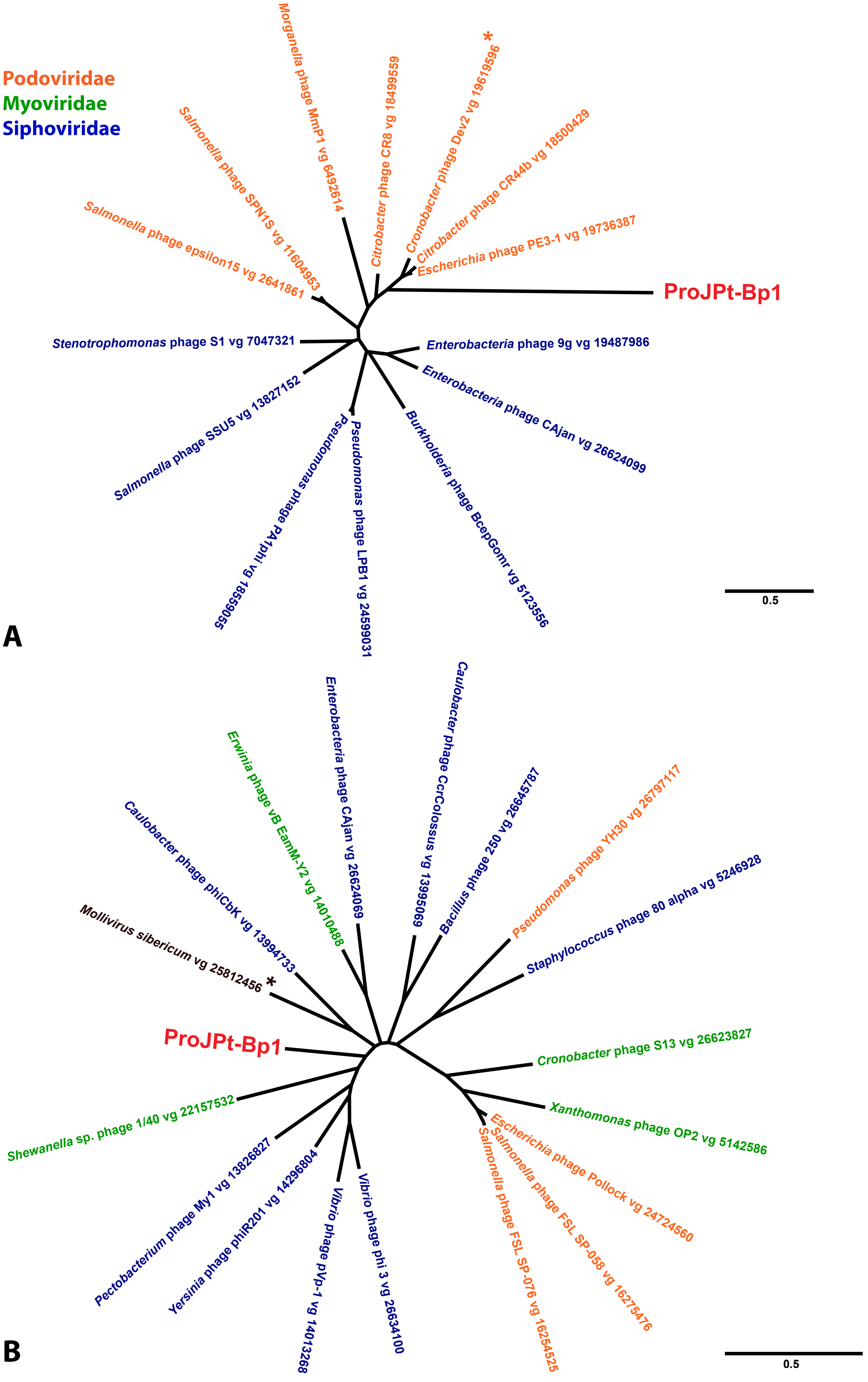

Podoviridae
Myoviridae
Siphoviridae
Morganella phage MmP1 vg 6492614
Citrobacter phage CR8 vg 18499559
Cronobacter phage Dev2 vg 19619596
*
Citrobacter phage CR44b vg 18500429
Escherichia phage PE3-1 vg 19736387
Salmonella phage SPN1S vg 11604953
Salmonella phage epsilon15 vg 2641861
ProJPt-Bp1
Stenotrophomonas phage S1 vg 7047321
Enterobacteria phage 9g vg 19487986
Enterobacteria phage CAjan vg 26624099
Salmonella phage SSU5 vg 13827152
Pseudomonas phage PA1phi vg 18659065
Pseudomonas phage LPB1 vg 24599031
Burkholderia phage BcepGomr vg 5123556
0.5
A
Caulobacter phage CcrColossus vg 13996069
Enterobacteria phage CAjan vg 26624069
Erwinia phage vB EamM-Y2 vg 14010488
Caulobacter phage phiCbK vg 13994733
Bacillus phage 250 vg 26645787
Pseudomonas phage YH30 vg 26797117
Staphylococcus phage 80 alpha vg 5246928
Mollivirus sibericum vg 25812456
*
ProJPt-Bp1
Cronobacter phage S13 vg 26623827
Xanthomonas phage OP2 vg 5142586
Shewanella sp. phage 1/40 vg 22157532
Pectobacterium phage My1 vg 13826827
Yersinia phage phiR201 vg 14296804
Vibrio phage pVp-1 vg 14013268
Vibrio phage phi 3 vg 26634100
Escherichia phage Pollock vg 24724560
Salmonella phage FSL SP-058 vg 16275476
Salmonella phage FSL SP-076 vg 16254525
0.5
B

Fig. S7. Neighbor-joining trees of phage-related genes based on deduced amino acid sequences. (A) Protein inside capsid D (111 amino acids); (B) dUTPase (114 amino acids). Sequences were aligned using ClustalW with a gap opening penalty of 10.0 and gap extension penalty of 0.2. The Dayhoff amino acid substitution model was used. An asterisk in panel (A) indicates the sequence from *Mollivirus sibericum*, the taxonomic assignment of which was unclear.

Table S1. Predicted genes on the ProJPt-Bp1 phage genome.

Gene_id*	start	stop	strand	predicted functions	Source**
PJPA_001	444	1	-	hypothetical protein	
PJPA_002	1040	444	-	hypothetical protein	
PJPA_003	1774	1049	-	hypothetical protein	
PJPA_004	2351	2010	-	hypothetical protein	
PJPA_005	2947	2399	-	hypothetical protein	
PJPA_tRNA	4241	4168	-	tRNA-Gln (CAG)	tRNA-scan
PJPA_006	5052	5672	+	hypothetical protein	
PJPA_007	5961	6296	+	hypothetical protein	
PJPA_008	6867	7556	+	hypothetical protein	
PJPA_009	7613	8083	+	hypothetical protein	
PJPA_010	8218	8805	+	hypothetical protein	
PJPA_011	8935	9546	+	ND2 superfamily protein	NCBI CDD
PJPA_012	9543	13154	+	hypothetical protein	
PJPA_013	13236	14219	+	hypothetical protein	
PJPA_014	14392	14715	+	hypothetical protein	
PJPA_015	14719	19752	+	Cadherin-like beta sandwich domain protein	NCBI CDD
PJPA_016	19949	21370	+	major outer envelope glycoprotein	NCBI CDD
PJPA_017	21348	24266	+	hypothetical protein	
PJPA_018	24273	27320	+	DUF4417-cotaining protien	NCBI CDD
PJPA_019	27317	30310	+	hypothetical protein	
PJPA_020	30609	32669	+	conserved hypothetical protein	
PJPA_021	32705	33322	+	hypothetical protein	
PJPA_022	33319	34041	+	hypothetical protein	
PJPA_023	34043	35188	+	hypothetical protein	
PJPA_024	35249	36301	+	hypothetical protein	
PJPA_025	36316	37275	+	hypothetical protein	
PJPA_026	37304	38458	+	VIP2; Actin-ADP-ribosylating toxin family protein	NCBI CDD
PJPA_027	38476	39948	+	putative structural protein	NR, refseq
PJPA_028	40082	40786	+	hypothetical protein	
PJPA_029	40783	41565	+	hypothetical protein	
PJPA_030	41569	42618	+	hypothetical protein	
PJPA_031	42615	43562	+	hypothetical protein	
PJPA_032	43562	46435	+	putative structural protein	NR, refseq
PJPA_033	46435	53412	+	hypothetical protein	
PJPA_034	53414	54700	+	hypothetical protein	
PJPA_035	54716	57367	+	hypothetical protein	
PJPA_036	57371	62218	+	Epstein-Barr virus nuclear antigen 3B domain protein	NCBI CDD
PJPA_037	62221	63048	+	hypothetical protein	
PJPA_038	63048	67547	+	hypothetical protein	
PJPA_039	67548	69068	+	hypothetical protein	
PJPA_040	69068	70708	+	hypothetical protein	
PJPA_041	70708	81153	+	putative structural protein	NR, refseq
PJPA_042	81153	85808	+	hypothetical protein	
PJPA_043	85805	86413	+	hypothetical protein	
PJPA_044	86410	92667	+	hypothetical protein	
PJPA_045	93306	94583	-	hypothetical protein	
PJPA_046	94941	94501	-	deoxyuridine 5'-triphosphate nucleotidohydrolase	NR, refseq

PJPA_047	96330	95398	-	hypothetical protein	
PJPA_048	96634	96287	-	solute carrier families 5 and 6-like protein	NCBI CDD
PJPA_049	97041	96631	-	phage protein inside capsid D	NR, refseq
PJPA_050	97633	97034	-	hypothetical protein	
PJPA_051	99091	98147	-	exonucleaase VIII	NR, refseq
PJPA_052	99407	99099	-	hypothetical protein	

* Red shows a tRNA gene; blue shows genes identified as phage-related in the METAVIR server.

**National Center for Biotechnology Information Conserved Domain Database (NCBI CDD); “non-redundant” database (NR); NCBI curated database (refseq).

Table S2. Codon usage (%) in the CfPt1-2 chromosome, ProJPt-1 chromosomes, and the ProJPt-Bp1 phage genome.

	U	Amino acid	CfPt1-2	ProJPt-1	ProJpt-Bp1	C	Amino acid	CfPt1-2	ProJPt-1	ProJpt-Bp1
U	UUC	Phe	20.0	12.2	53.7	UCA	Ser	18.7	26.9	32.1
	UUU	Phe	80.0	87.8	46.3	UCC	Ser	9.9	3.2	3.7
	UUA	Leu	36.2	50.7	29.7	UCG	Ser	7.7	4.0	5.1
	UUG	Leu	28.0	20.6	13.3	UCU	Ser	34.6	38.7	20.5
C	CUA	Leu	9.9	9.3	24.0	CCA	Pro	32.4	48.7	51.3
	CUC	Leu	4.1	1.7	8.6	CCC	Pro	11.0	1.6	1.4
	CUG	Leu	5.1	2.4	7.6	CCG	Pro	10.1	7.3	8.9
	CUU	Leu	16.7	15.3	16.7	CCU	Pro	46.5	42.4	38.5
A	AUA	Ile	33.2	35.7	49.9	ACA	Thr	38.1	44.8	48.8
	AUC	Ile	13.0	7.0	25.2	ACC	Thr	10.2	4.0	14.3
	AUU	Ile	53.8	57.3	24.9	ACG	Thr	9.5	5.5	11.4
	AUG	Met	100.0	100.0	100.0	ACU	Thr	42.2	45.7	25.6
G	GUA	Val	38.4	38.8	37.9	GCA	Ala	36.6	40.0	43.4
	GUC	Val	8.5	4.8	11.8	GCC	Ala	11.0	5.5	6.8
	GUG	Val	14.1	9.9	21.3	GCG	Ala	6.5	5.7	9.7
	GUU	Val	38.9	46.6	29.0	GCU	Ala	45.9	48.9	40.1

	A	Amino acid	CfPt1-2	ProJPt-1	ProJpt-Bp1	G	Amino acid	CfPt1-2	ProJPt-1	ProJpt-Bp1
U	UAC	Tyr	20.7	19.0	43.7	UGC	Cys	22.4	17.8	33.5
	UAU	Tyr	79.3	81.0	56.3	UGU	Cys	77.6	82.2	66.5
	UAA	STOP	53.3	62.2	50.9	UGA	STOP	27.9	23.7	29.8
	UAG	STOP	18.8	14.0	19.3	UGG	Trp	100.0	100.0	100.0
C	CAC	His	19.5	17.2	27.6	CGA	Arg	16.2	14.9	4.6
	CAU	His	80.5	82.8	72.4	CGC	Arg	7.3	6.4	6.8
	**CAA**	**Gln**	**77.9**	**84.9**	**47.4**	CGG	Arg	5.3	3.1	3.8
	**CAG**	**Gln**	**22.1**	**15.1**	**52.6**	CGU	Arg	30.6	33.4	30.0
A	AAC	Asn	18.8	14.1	46.1	AGC	Ser	6.1	4.2	17.7
	AAU	Asn	81.2	85.9	53.9	AGU	Ser	23.0	22.9	21.0
	AAA	Lys	78.2	88.2	33.5	AGA	Arg	30.3	34.6	34.0
	AAG	Lys	21.8	11.8	66.5	AGG	Arg	10.2	7.6	20.9
G	GAC	Asp	18.5	13.8	38.7	GGA	Gly	43.1	49.5	33.4
	GAU	Asp	81.5	86.2	61.3	GGC	Gly	8.3	7.9	16.2
	GAA	Glu	79.9	85.8	48.0	GGG	Gly	16.8	14.6	11.9
	GAG	Glu	20.1	14.2	52.0	GGU	Gly	31.9	28.0	38.4

Blue indicates codons of which corresponding tRNA genes are present in both ProJPt-1 and CfPt1-2 genomes; green indicates those only in ProJPt-1; orange indicates those only in CfPt1-2; yellow indicates those in both CfPt1-2 and ProJPt-Bp1 (absent in ProJPt-1).
